# The mediating role of delivery mode on postpartum maternal depression and mother-infant bonding

**DOI:** 10.12669/pjms.41.6.11759

**Published:** 2025-06

**Authors:** Kamber Kasali, Gamze Nur Cimilli Senocak, Aysenur Dostbil, Emsal Pinar Topdagi Yilmaz, Agah Abdullah Kahramanlar, Sabri Selcuk Atamanalp

**Affiliations:** 1 Kamber Kasali Assistant Professor, Department of Biostatistics, Faculty of Medicine, Ataturk University, Erzurum, Turkiye; 2 Gamze Nur Cimilli Senocak, MD Associate Professor, Department of Obstetrics and Gynecology, Faculty of Medicine, Ataturk University, Erzurum, Turkiye; 3 Aysenur Dostbil, MD Professor, Department of Anesthesiology and Reanimation, Faculty of Medicine, Ataturk University, Erzurum, Turkiye; 4 Emsal Pinar Topdagi Yilmaz, MD Associate Professor, Department of Obstetrics and Gynecology, Faculty of Medicine, Ataturk University, Erzurum, Turkiye; 5 Agah Abdullah Kahramanlar, MD Specialist, Clinic of Anesthesiology and Reanimation, Erzurum City Hospital, Erzurum, Turkiye; 6 Sabri Selcuk Atamanalp, MD Professor, Department of General Surgery, Faculty of Medicine, Ataturk University, Erzurum, Turkiye

**Keywords:** Cesarean delivery, Mother-infant bonding, Mediating role, Postpartum depression, Vaginal delivery

## Abstract

**Objectives:**

The mother-infant relationship is a magical process that begins with conception and strengthens when the mother feels the baby’s movements. This special bond, formed during pregnancy, is shaped by the parent’s emotions, perceptions, and behaviors toward the fetus. Research shows that heightened maternal anxiety during pregnancy and childbirth affects maternal behavior and impairs mother-infant bonding. While studies suggest that delivery mode influences maternal anxiety and postpartum depression, results often vary due to group differences and confounding factors. Although delivery mode may impact postpartum depression and mother-infant bonding, direct research on its specific effects remains limited.

**Methods:**

In this prospective study, a structured questionnaire was administered to 208 women in six months period between March, 2024 and September, 2024 in Ataturk University Faculty of Medicine Research Hospital. The survey aimed to gather participants’ demographic information and assess their mother-infant bonding and postpartum depression statuses. The Mother-infant bonding scale (MIBS) and the Edinburgh postpartum depression scale (EPDS) were used for data collection.

**Results:**

The analysis determined that delivery mode did not significantly affect the EPDS total score and MIBS total score relation. The indirect effect was calculated as 0.001±0.001 (CI:-0.003 to 0.001, β =-0.002, p = 0.635), the direct effect as -0.170±0.228 (CI:-0.618 to 0.277, β =-0.127, p=0.063), and the total effect as -0.036±0.019 (CI:-0.074 to 0.001, β=-0.129, p = 0.059).

**Conclusion:**

This study showed that mode of delivery did not affect the postnatal maternal depression on mother-infant bonding.

## INTRODUCTION

The mother-infant relationship is a profound process that begins with conception and deepens as the mother perceives the baby’s movements. This unique bond, forming during pregnancy, is shaped by the parent’s emotions, perceptions, and specific behaviors toward the fetus.^1^ Numerous biochemical processes during pregnancy influence the development of this bond, along with factors such as the delivery process, postpartum experiences, and demographic, perinatal, and psychological factors.^1^,^2^ The bond between mother and infant acts an important part in the baby’s cognitive and emotional status in life.^1^

Studies indicate that maternal anxiety levels during pregnancy and childbirth can affect maternal behaviors toward the baby and lead to bonding problems.^3^,^4^ Severe anxiety can lead to postpartum depression, disrupting the mother-infant relationship and adversely affecting family dynamics, breastfeeding behaviors, and the baby’s psychosocial development.^5^,^6^ While the relationships between postpartum depression and mother-infant bonding, as well as postpartum depression and delivery mode, are well-researched, postpartum depression is shown to have a direct effectiveness on mother-infant bonding.^5^,^7^

The significance of antenatal anxiety and postpartum depression during the delivery process is linked to various factors, including delivery mode,^8^,^9^ whether the baby stays with the mother or in the neonatal intensive care unit,^10^ absence of partner or social accompaniment, unplanned or undesired pregnancies, and presence of complications or pregnancy loss in current or past pregnancies.^11^ Despite numerous studies suggesting that delivery mode can affect maternal anxiety levels and contribute to postpartum depression, the findings are inconsistent and contradictory due to differences in group characteristics and confounding factors.^8^,^9^

Although delivery mode is thought to significantly impact both postpartum depression and mother-infant bonding, there is not enough studies directly exploring the effect of delivery mode on mother-infant bonding.^9^ This research evaluates the effects of delivery mode on postpartum depression and mother-infant bonding in women who have given birth, and examines the relationship between these factors. Our goal was to evaluate deeply the importance of delivery mode in terms of mother-infant bonding and to determine whether the impact of delivery mode on bonding is mediated by its influence on maternal anxiety and depression. This study is the first in the literature evaluating the mediating effects of delivery mode on postpartum depression and mother-infant bonding relation.

## METHODS

In this prospective study, a structured questionnaire was administered to 208 women in 6-month period between March 2024 and September 2024 in Ataturk University Faculty of Medicine Research Hospital. The questionnaire was designed to collect participants’ demographic information and assess their mother-infant bonding and postpartum depression statuses. Responses were collected from participants who provided voluntary consent to join the study. After all data were collected, statistical analyses were performed to evaluate the findings.

### Ethical Approval:

An approval was obtained from the ethics committee of Ataturk University Faculty of Medicine (Ref. No. 124; dated Feb. 21, 2024). Informed consents were obtained from all individuals.

### Measurement Tools:

### Mother-Infant Bonding Scale (MIBS):

The scale is designed to evaluate the emotional bond established by mothers with their infants postpartum. It aims to measure maternal emotions toward the infant and analyze bonding levels. The scale, which links maternal mood to emotional bonding, was developed by Taylor et al.^12^ and is originally called the ‘Mother-to-infant bonding scale’. The Turkish version, adapted by Aydemir Karakulak and Alparslan,^13^ was culturally validated and its reliability tested. MIBS is an 8-item, 4-point Likert-type scale with a total score range of 0-24. Higher scores indicate stronger mother-infant bonding. One subdimension, **positive emotions,** evaluates expressions of love, joy, and protectiveness through items 1, 4, and 6, which are scored as 0, 1, 2, or 3. The other subdimension, **negative emotions,** assesses feelings like dislike, disappointment, anger, and frustration through items 2, 3, 5, 7, and 8, which are reverse-scored (3, 2, 1, 0). Factor analysis confirmed the scale’s construct validity by identifying two core factors: positive and negative bonding emotions. The reliability analysis yielded a Cronbach’s alpha coefficient of 0.69 for mothers and 0.79 for fathers, indicating satisfactory reliability levels for the scale.

### Edinburgh Postpartum Depression Scale (EPDS):

The EPDS was developed by Cox et al.^14^ in 1987 and is designed to assess the hazard of depression in women during the postpartum period. The scale’s Cronbach’s alpha coefficient was determined as 0.87, signifying high reliability. Its validity and reliability in Turkish were tested by Engindeniz et al.^15^ in 1996. EPDS is a self-assessment, Likert-type scale consisting of 10 items. It is specifically designed to measure mood changes in patients. Each item is scored differently, and the scale provides a total score ranging from 0 to 30, with higher scores indicating a higher risk of postpartum depression. Items 1, 2, and 4 are scored as ‘0-1-2-3’, while items 3, 5, 6, 7, 8, 9, and 10 are reverse-scored as ‘3-2-1-0’. The cutoff point for identifying cases is 13 points. In this study, mothers scoring 13 or higher were classified into the case group. The Cronbach’s alpha coefficient for this study was calculated as 0.89, demonstrating excellent reliability for the scale.

### Statistical Analysis:

The data were presented as mean, median, standard deviation, minimum, maximum, percentage, and frequency. The normality of distribution for quantitative variables was evaluated using the Shapiro-Wilk tests. For comparisons between two independent groups, Mann-Whitney U test was applied because the normality assumption was not met. A statistical technique called mediator analysis is used to determine whether a third variable mediates the relationship between an independent variable and a dependent variable. Our study used mediation model analysis to look at how the mode of delivery affected postpartum maternal depression and mother-infant attachment. The structural equation modeling bootstrapping method was utilized to assess the significance of the indirect effects of the scales tested in the model. Path coefficients (β) were calculated for the model, and multivariate normality was evaluated using the Mardia test. All analyses were conducted using IBM SPSS 20 and JAMOVI 2.2.2 statistical software, with statistical significance level was accepted as p < 0.05.

## RESULTS

A total of 208 women were included in this study. The demographic and health-related features are highlighted in Table-I. The findings underscore that 80.30% of the participants gave birth via cesarean section, while the rate of vaginal deliveries was 19.20%. Regarding marital status, most participants (99.00%) were married. In terms of education level, 38.50% of participants were university graduates, while 30.80% had completed primary school. Employment status showed that most participants (76.00%) were employed, whereas 24.03% were unemployed. Most participants (94.20%) were reported as nonsmokers. Data on health conditions revealed that 24.00% of participants indicated having a chronic illness, and 41.80% reported having undergone surgery before. For psychiatric health, 5.80% of participants had a history of psychiatric illness, and 7.70% had sought medical consultation for psychiatric conditions.

**Table-I:** Demographic characteristics of the participants.

		Count	Column N %
Delivery mode	Cesarean	167	80.30
	Vaginal	40	19.20
	Painless	1	0.50
Marital status	Married	206	99.00
	Divorced	2	1.00
Education level	Illiterate	2	1.00
	Literate	7	3.40
	Primary school	64	30.80
	High school	55	26.40
	University	80	38.50
Employment status	Employed	158	76.00
	Unemployed	50	24.03
Smoking	Yes	12	5.80
	No	196	94.20
Alcohol using	No	208	100.00
History of curettage	0	175	84.10
	1	23	11.10
	2	9	4.30
	3	1	0.50
Chronic illness	Yes	50	24.00
	No	158	76.00
Previous surgery	Yes	87	41.80
	No	121	58.20
Previous hospitalization	Yes	107	51.70
	No	100	48.30
Regular medication	Yes	40	19.20
	No	168	80.80
Psychiatric disorder	Yes	12	5.80
	No	196	94.20
Psychiatric consultation	Yes	16	7.70
	No	192	92.30

As seen in Table-II, the statistical data obtained from the analyses are as follows: The mean total score for EPDS was 5.00±5.00, while the mean total score for MIBS was 15.25±1.38. The average age was 31.00±6.00 years. The average number of cigarette packs smoked was 5.00±3.00, and the mean pregnancy number was 3.00±2.00. The mean number of miscarriages was 0.00±1.00. As shown in the Table-III, the average age was 31±6 years for both cesarean and vaginal delivery groups. The difference in average age between the delivery modes was not statistically significant (Z=-0.107, p=0.914). In terms of EPDS total scores, the average score for participants who delivered via cesarean section was 5±5, while the average score for those who delivered vaginally was 6±5. The median score for groups was 4, 5, and the difference in EPDS scores between the groups was not statistically significant (Z=-1.028, p=0.304). For MIBS total scores, the average for cesarean deliveries was 15.28±1.33, while the average for vaginal deliveries was 15.25±1.5. No statistically significant difference was observed in this case either (Z=-0.144, p=0.885).

**Table-II:** Descriptive statistics of variables.

	Mean	Standard deviation	Median	Minimum	Maximum
Age	31.00	6.00	30	18	46
Number of cigarette packs	5.00	3.00	4	1	12
Number of pregnancies	3.00	2.00	2	1	10
Number of miscarriages	0.00	1.00	0	0	6
EPDS total score	5.00	5.00	4	0	21
MIBS total score	15.25	1.38	15	9	20

EPDS: Edinburgh postpartum depression scale, MIBS: Mother-infant bonding scale.

**Table-III:** Comparison of age, EPDS total score, and MIBS total score by delivery mode.

	Delivery mode											
	Cesarean delivery					Vaginal delivery						
	Mean	SD	Med	Min	Max	Me	SD	Med	Min	Max	Z	p
Age	31	6	30	19	46	31	6	30	18	41	-0.107	0.914
EPDS total Score	5.00	5.00	4	0	21	6.00	5.00	5	0	17	-1.028	0.304
MIBS total score	15.28	1.33	15	9	20	15.25	1.50	15	12	19	-0.144	0.885


Me: Mean, SD: Standard deviation, Z: Mann Whitney u test, EPDS: Edinburgh postpartum depression scale, MIBS: Mother-infant bonding scale.

The mediating role of delivery mode in the EPDS total score and MIBS total score relation was analyzed using the bootstrapping method (Figure-I). The indirect effect of delivery mode on MIBS total score via EPDS total score was estimated as 0.001±0.001, with a confidence interval ranging (CI:-0.003 to 0.001), showing no statistical significance (β=-0.002, z=-0.475, p=0.635). Component effects revealed that the relationship between EPDS total score and delivery mode was 0.003±0.005 (CI:-0.007 to 0.015) and not statistically significant (β=0.042, z=0.615, p=0.538). The effect of delivery mode on MIBS total score was -0.170±0.228 (CI:-0.618 to 0.277), which was also not statistically significant (β=-0.051, z=-0.746, p=0.456). Direct effect analysis showed that the estimated direct effect between EPDS total score and MIBS total score was -0.035±0.019 (CI -0.073 to 0.01) and not statistically significant (β=-0.127, z=-1.859, p=0.063). Similarly, the total effect was calculated as -0.036±0.019 (CI:-0.074 to 0.001) and was also not significant (β=-0.129, z=-1.886, p = 0.059). These results demonstrate that delivery mode does not have a significant mediating role in the EPDS total score and MIBS total score relation. Both the indirect and direct effects were not statistically significant (Table-IV).

**Fig.1:**
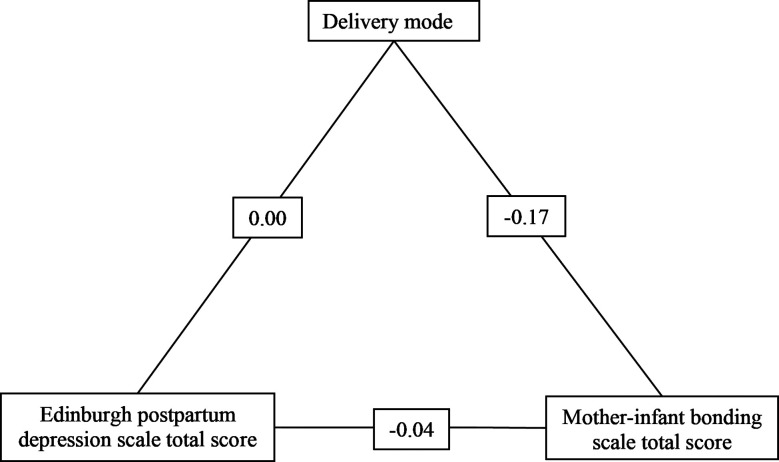
Mediation path diagram

**Table-IV:** Mediation analysis results of EPDS total score, delivery mode, and MIBS total score.

Indirect and total effects								
				95% C.I. (a)				
Type	Effect	Estimate	SE	Lower	Upper	β	z	p
Indirect	EPDS TS ⇒ DM ⇒ MIBS TS	0.001	0.0012	-0.003	0.001	-0.002	-0.475	0.635
Component	EPDS TS ⇒ DM	0.003	0.0058	-0.007	0.015	0.042	0.615	0.538
	DM ⇒ MIBS TS	-0.170	0.2287	-0.618	0.277	-0.051	-0.746	0.456
Direct	EPDS TS ⇒ MIBS TS	-0.035	0.0192	-0.073	0.001	-0.127	-1.859	0.063
Total	EPDS TS ⇒ MIBS TS	-0.036	0.0192	-0.074	0.001	-0.129	-1.886	0.059

SE: Standard error, EPDS TS: Edinburgh postpartum depression scale total score, DM: Delivery mode, MIBS TS: Mother-infant bonding scale total score. Note: Confidence intervals computed with method: Standard (Delta method), Note: Betas are completely standardized effect sizes. Indirect effect: Postnatal depression affects mother-infant attachment through mode of delivery, Direct effect: The effect of postnatal depression on mother-infant attachment.

## DISCUSSION

This study presents a direct relation between postpartum depression and the extent of mother-infant bonding, aligning with findings in the existing literature. It is evident that postpartum depression and anxiety disrupt mother-infant bonding. Furthermore, the findings support the assertion that delivery mode does not mediate the maternal postpartum anxiety or depression (EPDS criteria) and mother-infant bonding (MIBS criteria) relationship. These results align with some findings in the literature while also presenting notable differences.

The impact of cesarean delivery on postpartum anxiety and depression has been a frequent topic of discussion in the literature. Clout and Brown^16^ found that cesarean delivery was associated with postpartum depression, anxiety, and stress levels in 2015. However, this relationship lost its significance when antenatal stress levels were controlled for, suggesting that delivery mode may not be an independent factor. Although Clout and Brown^16^ did not assess mother-infant bonding, this is consistent with our study’s findings, which also propose that delivery mode is not an independent factor. In 2016, Bell et al.^17^ investigated the effects of mode of delivery on postnatal depression and anxiety. The study presented that mothers’ perceptions of their last birth experience had a direct effect on postnatal anxiety levels. However, there was no direct relationship between delivery mode itself and postpartum depression or anxiety in their study. These findings align with our study, highlighting that postpartum psychological outcomes are influenced more by perceptual and environmental factors than by delivery mode alone. Notably, Bell et al.^17^ also did not evaluate mother-infant bonding.

Chen et al.^18^ found that cesarean delivery significantly increased postpartum stress levels but had no significant effect on anxiety or depression in 2017. This indicates that while delivery mode, particularly cesarean delivery, may be associated with certain psychological outcomes, it does not establish a general cause-and-effect relationship. As in the studies mentioned above, Chen et al.^18^ did not investigate the role of delivery mode in mother-infant bonding. Finally, a meta-analysis by Moameri et al.^19^ suggested that cesarean delivery could be a mild risk factor for postpartum depression in 2019. However, our study found no apparent effect of delivery mode on mother-infant bonding or maternal anxiety. This discrepancy may be due to differences in methodological approaches or demographic characteristics of the study samples. Additionally, Moameri et al.^19^ did not explore mother-infant bonding.

In the literature, the relationship between mother-infant bonding and postpartum depression has been extensively studied. However, in our study, we evaluated the effects of antenatal anxiety, including anxiety and stress factors developing during both the pregnancy and postpartum periods, on mother-infant bonding, as well as how delivery mode influences these factors and whether it directly or indirectly affects bonding through anxiety levels. In the literature, only one study examining these three factors together was found.^9^ However, it was handled differently from our study in some aspects.^9^ This study divided participants into two groups, cesarean and vaginal delivery, and compared postpartum depression and mother-infant bonding between these groups using multiple linear regression analysis. The study separately examined the effects of delivery mode and postpartum depression levels on mother-infant bonding. In contrast, our study also investigated whether delivery mode had an indirect effect on mother-infant bonding through its mediating role. In the study by Topbas Selcuki NF et al.,^9^ it was found that delivery mode did not affect mother-infant bonding, but postpartum depression negatively influenced bonding. In our study, however, it was concluded that delivery mode had no effect on mother-infant bonding and that delivery mode did not play a mediating role in the effect of postpartum depression on mother-infant bonding.

In our literature review, we did not find any article investigating the effect of mode of delivery on mother-infant bonding through its effect on maternal anxiety. This is the first study in the literature to look at mother-infant bonding from this perspective. The strength of this study is that while it investigates the effect of mode of delivery on mother-infant bonding, it also investigates the way in which it may have had this effect and whether it may have done so by affecting maternal anxiety. Special statistical method was used for this evaluation. In order to contribute to the literature on this subject, more comprehensive studies should be conducted to investigate the effect of mode of delivery on mother-infant bonding, and studies should be planned to investigate which factors such as anxiety level may have influenced this effect.

### Limitations:

In our study, we used the EPDS criteria as postpartum depression questionnaire, but mother-baby bonding is also related to pregnancy and the birth process, means antenatal anxiety, and an additional questionnaire might have been used to assess the level of depression or anxiety during pregnancy and birth process. Another limitation is all patients in the study group were evaluated at a very early stage of mother-infant bonding. To better evaluate mother-infant bonding, re-evaluating it at a later period could have contributed to the study. Since our hospital is a tertiary hospital that receives referrals from distant provinces, it was difficult to reach the patients after discharge, and the late mother-infant bonding could not be evaluated in the study.

## CONCLUSIONS

This study found that delivery mode (cesarean or vaginal delivery) does not have a significant effect on postpartum maternal depression or mother-infant bonding. This study uniquely evaluates the mediating role of delivery mode in the postpartum depression and mother-infant bonding relation. Compared to previous research, our findings indicate that the psychological outcomes of delivery mode are indirect and influenced by individual and environmental factors. Future studies are recommended to explore the psychological effects of delivery mode using more comprehensive models that incorporate individual, social, and environmental factors.

### Authors’ Contributions:

**KK:** Project development, Data collection, Manuscript writing, Manuscript editing, and Data analysis.

**GNCS, EPTY, AD:** Project development, Data collection, Manuscript writing, and Manuscript editing.

**AAK:** Literature search, Data collection.

**SSA:** Project development, Critical Review and Manuscript writing. He is also responsible and accountable for the accuracy and integrity of the work.

## References

[1] Pisoni C, Garofoli F, Tzialla C, Orcesi S, Spinillo A, Politi P (2014). Risk and protective factors in maternal-fetal attachment development. Early Hum Dev.

[2] Rolle L, Giordano M, Santoniccolo F, Trombetta T (2020). Prenatal attachment and perinatal depression:A systematic review. Int J Environ Res Public Health.

[3] O'Dea GA, Youssef GJ, Hagg LJ, Francis LM, Spry EA, Rossen L (2023). Associations between maternal psychological distress and mother-infant bonding:a systematic review and meta-analysis. Arch Womens Ment Health.

[4] Brassel A, Townsend ML, Pickard JA, Grenyer BFS (2020). Maternal perinatal mental health:Associations with bonding mindfulness, and self-criticism at 18 months'postpartum. Infant Ment Health J.

[5] Meky HK, Shaaban MM, Ahmed MR, Mohammed TY (2020). Prevalence of postpartum depression regarding mode of delivery:a cross-sectional study. Matern Fetal Neonatal Med.

[6] Johnson AR, Edwin S, Joachim N, Mathew G, Ajay S, Joseph B (2015). Postnatal depression among women availing maternal health services in a rural hospital in South India. Pak J Med Sci.

[7] Dubber S, Reck C, Müller M, Gawlik S (2015). Postpartum bonding:the role of perinatal depression anxiety and maternal-fetal bonding during pregnancy. Arch Womens Ment Health.

[8] Hughes C, Foley S, Devine RT, Ribner A, Kyriakou L, Boddington L (2020). Worrying in the wings?Negative emotional birth memories in mothers and fathers show similar associations with perinatal mood disturbance and delivery mode. Arch Womens Ment Health.

[9] Topbas Selcuki NF, Yalcin Bahat P, Turan G, Aksoy U, Bagci K, Ozdemir I (2022). Postpartum evaluation of the role of maternal characteristics and mode of delivery on maternal attachment anxiety and depression;a study conducted in Turkey'. Acta Biomed.

[10] Mizrak B, Deniz AO, Acikgoz A (2015). Anxiety levels of mothers with newborns in a Neonatal Intensive Care Unit in Turkey. Pak J Med Sci.

[11] Biaggi A, Conroy S, Pawlby S, Pariante CM (2016). Identifying the women at risk of antenatal anxiety and depression:A systematic review. J Affect Disord.

[12] Taylor A, Atkins R, Kumar R, Adams D, Glover V (2005). A new Mother-to-Infant Bonding Scale:Links with early maternal mood. Arch Womens Ment Health.

[13] Aydemir Karakulak H, Alparslan O (2016). Anne-bebek bağlanma ölçeğinin Türk Toplumu'na uyarlanması:Aydın örneği. Çagdas Tip Derg.

[14] Cox JL, Holden JM, Sagovsky R Detection of postnatal depression:Development of the 10-item Edinburgh Postnatal Depression Scale. Br J Psychiatry.

[15] Engindeniz AN, Küey L, Kültür S (1996). Edinburgh doğum sonrasıdepresyon ölçeği Türkçe formu geçerlilik ve güvenilirlik çalışması. Bahar Sempozyumları.

[16] Clout D, Brown R, Sociodemographic pregnancy (2015). obstetric and postnatal predictors of postpartum stress anxiety and depression in new mothers. J Affective Dis.

[17] Bell A, Carter C, Davis J, Golding J, Adejumo O, Pyra M (2016). Childbirth and symptoms of postpartum depression and anxiety:a prospective birth cohort study. Arch Womens Ment Health.

[18] Chen H, Lai J, Hwang S, Huang N, Chou Y, Chien L (2017). Understanding the relationship between cesarean birth and stress anxiety, and depression after childbirth:A nationwide cohort study. Birth.

[19] Moameri H, Ostadghaderi M, Khatooni E, Doosti-Irani A (2019). Association of postpartum depression and cesarean section:A systematic review and meta-analysis. Clin Epidemiol Global Health.

